# Progress in Surgery

**Published:** 1898-11-12

**Authors:** 


### Progress in Surgery.

### SURGERY OF THE BRAIN.

**The Value of Cranial Percussion of the Skull in the Diagnosis of Surgical Affections of the Brain.**—Although the importance of observing the percussion note of the skull in the diagnosis of intracranial conditions has for a number of years been insisted upon by several writers, notably Macewen, Suckling, and Bruns, its use has not become general with surgeons. This has probably been due to the fact that we had no data as to the normal percussion note of the skull, and the commoner variations which occur in healthy individuals. A recent inquiry by Paoli and Mori[1] helps to clear the ground in this direction. These observers have found that it is necessary to shave the head, and to percuss with the finger directly on the surface to obtain reliable results. A high degree of resonance, increased by opening the mouth, is the characteristic note of the normal skull, but this varies in degree according to the area percussed and the age and sex of the subject examined. The density of the skull cap also influences the note, as might be expected. The dullest note is obtained over the frontal sinuses and mastoid processes, especially in young children.

The head is divided into three synethical areas—frontal, parietal, and occipital, and the note of corresponding areas on the two sides are compared.

In boys below ten years of age the temporal and parietal regions yield a much more resonant note than the frontal and occipital areas. In rickety children the resonance is still greater, a cracked-pot sound being not uncommon. In adult women the note resembles that of the child, but in very old women the resonance diminishes. The opposite is the case with men. In adults the average note is duller than in childhood, but becomes more resonant as age advances.

Experiments were performed on the cadaver by injecting various substances, such as yolk of egg, blood and water, through small openings, and observing the effect upon the percussion note. It was found on opening the skulls that the area of increased dulness corresponded almost exactly to the distribution of the foreign substance and varied directly with the thickness of it. To avoid any fallacy which might arise through making an opening in the skull, another series of observations were made, in which the foreign substance was rejected through the occipital foramen. The results confirmed those obtained by the first set.

Several observations were also made upon patients suffering from intra-cranial conditions. For example, in the case of a young man who suffered from epilepsy following on a complicated fracture of the frontal bone, an area of dulness was made out all along the line of the fracture. On operating, it was found that the dura was much thickened, and that an old hæmatoma existed which corresponded exactly to the area of dulness. In another case of injury to the head with effusion of blood, producing paralysis of the right arm and aphasia, as the effusion became absorbed, and the symptoms passed off, the marked dulness which existed gradually disappeared, and was replaced by the normal resonance.

In a case of tuberculous meningitis, the writer diagnosed by the dulness being more marked on the right than on the left side, that the pathological process was correspondingly distributed, and post-mortem examination confirmed this opinion. In another case the same phenomena were observed, but recovery took place, and with it the note of the two sides became equalised.

Other observations are quoted which go to prove the value of this hitherto too much neglected diagnostic measure.

**Bullets in the Brain and the Roentgen Rays.**—The possibility of accurately localising bullets which have entered the skull by means of the Roentgen rays is a distinct advance in cerebral diagnosis, but it is apt to give a stimulus to zealous operators to remove bullets which it would be safer to leave alone. Von Bergmann[2] clearly condemns operative interference in such cases. He quotes 32 cases of bullet wounds of the brain which have come under his own observation. Of these, eight were rapidly fatal; two developed frontal abscess, which proved fatal; other two succumbed to suppurative meningitis, and one is not accounted for. The remaining 19 patients recovered, and remained well, thus showing that the removal of bullets is by no means a necessity. He refers to two cases in which the bullet was accurately localised by aid of the X rays. In one case it was removed from the white matter of the occipital lobe, and the patient recovered from marked eye symptoms; in the other the bullet was in the posterior limb of the internal capsule, and caused great pain in the head. Although anxious to have it removed, he was dissuaded, and eventually recovered. Bergmann advocates leaving these cases alone, avoiding searching examinations, and adopting rigid precautions against infection. When the bullet is in the bone or membranes it should be removed, of course.

Braatz,[3] on the other hand, is encouraged by the success of an operation, by which he removed a bullet from the brain, after localising it by means of the Roentgen rays.

**Osteoplastic Craniectomy.**—The advantages of reflecting a flap comprising bone and scalp in extensive operations on the brain are acknowledged, and many measures have been devised to effect this. One of the latest is that of Gigli,[4] which consists in passing fine wire saws through four small openings in the skull, and cutting from within outward a rectangular flap of bone, the lower side of which is simply broken across. The saws are introduced by means of a stillete of whalebone, and can be applied so as to give a bevelled surface, which prevents the flap falling in upon the brain when replaced. Keen[5] is satisfied with this apparatus, which, he thinks, has the following advantages: (1) The ease with which the edges of the bone can be bevelled, and the infinitesimally small loss of bone on account of the thinness of the saw; (2) the fact that in a thick skull the fourth side of the flap may be partially sawn through before being broken; and (3) the avoidance of jarring (which, by the way, he considers a theoretical objection to the chisel and mallet). Buchanan,[6] of Pittsburg, Pa., had devised and used an apparatus very similar to this before he knew of Gigli's using it.

[1] Il Policlinico, Feb. 15, 1898; and La Semaine Med., Mar. 26, 1898. [2] Berl. Klin. Woch., May 2, 1898. [3] Centralbl. f. Chir., 1898, No. 1. [4] Centralbl. f. Chir., April 23, 1898. [5] Philadelphia Med. Jour., Jan., 1898. [6] Med. Rec., June 4, 1898.

